# Safety and Efficacy of Stereotactic Arrhythmia Radioablation for the Treatment of Ventricular Tachycardia: A Systematic Review

**DOI:** 10.3389/fcvm.2022.870001

**Published:** 2022-08-22

**Authors:** Giovanni Volpato, Paolo Compagnucci, Laura Cipolletta, Quintino Parisi, Yari Valeri, Laura Carboni, Andrea Giovagnoni, Antonio Dello Russo, Michela Casella

**Affiliations:** ^1^Cardiology and Arrhythmology Clinic, University Hospital “Ospedali Riuniti”, Ancona, Italy; ^2^Department of Biomedical Sciences and Public Health, Marche Polytechnic University, Ancona, Italy; ^3^Cardiac Surgery Anesthesia and Critical Care Unit, University Hospital “Ospedali Riuniti”, Ancona, Italy; ^4^Department of Radiology, University Hospital “Ospedali Riuniti”, Ancona, Italy; ^5^Department of Clinical, Special and Dental Sciences, Marche Polytechnic University, Ancona, Italy

**Keywords:** radioablation, arrhythmia, electric storm, ventricular tachycardia, systematic review

## Abstract

Catheter ablation (CA) is a fundamental therapeutic option for the treatment of recurrent ventricular arrhythmias. Notwithstanding the tremendous improvements in the available technology and the increasing amount of evidence in support of CA, in some patients the procedure fails, or is absolutely contraindicated due to technical or clinical issues. In these cases, the clinical management of patients is highly challenging, and mainly involves antiarrhythmic drugs escalation. Over the last 5 years, stereotactic arrhythmia radioablation (STAR) has been introduced into clinical practice, with several small studies reporting favorable arrhythmia-free outcomes, without severe side effects at a short to mid-term follow-up. In the present systematic review, we provide an overview of the available studies on stereotactic arrhythmia radioablation, by describing the potential indications and technical aspects of this promising therapy.

## Introduction

Currently, catheter ablation (CA) is the treatment of choice for drug-refractory macroreentrant ventricular arrhythmias (1). The aim of CA is the elimination of clinical ventricular tachycardias (VT) and the modification of the myocardial substrate by abolishing areas displaying abnormal fragmented/late electrograms, which highlight the presence of viable slow-conducting myocardial fibers often interspersed with fibrous tissue and potentially responsible for further reentrant circuits (2).

In the last decade, a new form of non-invasive ablation using radiotherapy has been introduced in the field of clinical cardiac electrophysiology. Stereotactic radiosurgery is a form of radiation therapy in which high-dose ionizing radiations affect a localized part of tissue (3). In comparison to conventional radiotherapy (RT) with linear accelerators, stereotactic RT delivers radiation in the target tissue from different trajectories: this technique allows to deliver high dose of ionizing particles in the target zone and to minimize irradiation in the surrounding tissues. The technique was first introduced in the 1950s by the Swedish neurosurgeon Lars Leksell for the treatment of intracranial tumors (4).

The first preclinical studies of stereotactic radiosurgery for the treatment of arrhythmias in guinea pigs dates back to 2010 (5). In the first stereotactic radioablation procedures, lesions were created inside the atrium for blocking the cavotricuspid isthmus, the atrioventricular node and the junction between the pulmonary veins and the left atrium. To obtain an effective ablation it was necessary to perform deliveries with a single high intensity radiation dose, ranging between 40 and 70 Gy. Higher dose of radiation is necessary to create lesion in the conduction system (AV nodal ablation), whereas ventricular and atrial walls are more sensitive to radiation and 30–40 Gy dose seems to be effective. The time window required for maximal clinical efficacy ranged between 35 and 50 days, which are required for the formation of connective tissue.

## Materials and Methods

### Data Sources and Search

We performed a comprehensive search in MEDLINE using keywords related to STAR and VT. The search was update on January 27, 2022, and was limited to human studies in peer reviewed journals in English language. This search was conducted using the terms “(radiosurgery OR radioablation OR STAR OR SBRT) AND (ventricular tachycardia OR ventricular tachyarrhythmia).”

### Study Selection

This manuscript has been prepared using Preferred Reporting Items for Systematic Reviews and Meta-Analysis guidelines for reporting (PRISMA) (6).

**FIGURE 1:**
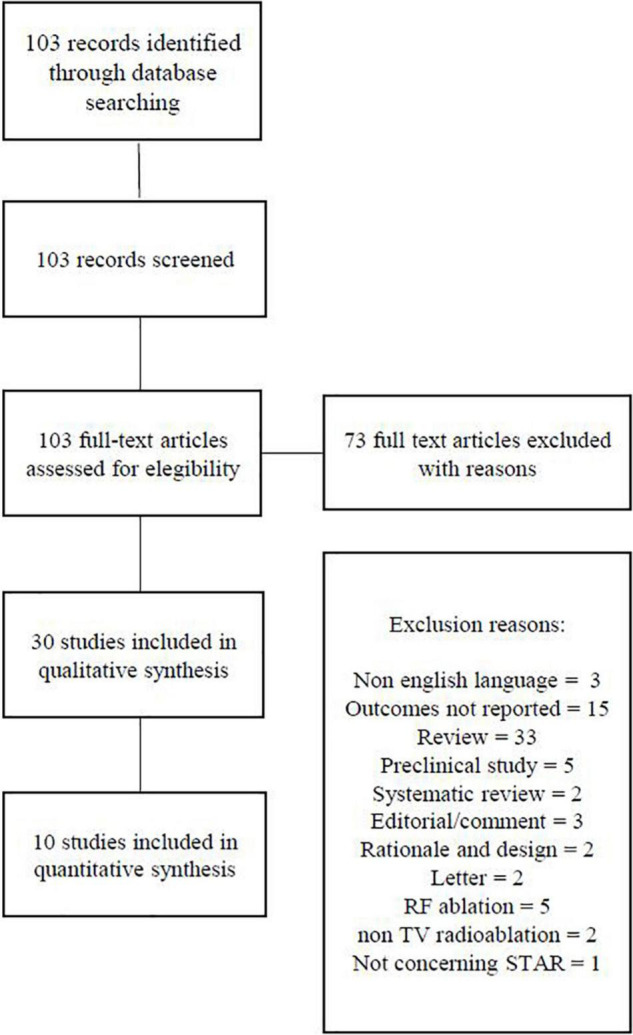
PRISMA flow diagram.

We identified 103 articles, out of which 30 full-text articles were reviewed for possible inclusion (Figure 1).

Two reviewers independently screened titles for inclusion criteria and then examined the full text of potentially suitable publications. All original studies of all designs about STAR to treat VT reporting outcome and safety data were included. Being reports of single cases, 20 studies were excluded from the review. The studies have to fulfill the following criteria to be included in the analysis: enrolling two or more patients, reporting a primary effectiveness outcome (defined as post-procedure sustained VT burden) as well as safety data (defined as adverse events related to the procedure).

### Data Extraction and Quality Assessment

Two reviewers independently adjudicated study quality and carried out the risk-of-bias assessment of eligible publications. Data were extracted using standardized protocol and reporting forms.

### Data Analysis, Synthesis, and Statistics

The studies included in this review are heterogeneous in terms of outcomes, patient characteristics, and indications for the procedure, so it was not possible to perform a meta-analysis.

The effectiveness of the procedure was related to reduction of VT burden at follow up. Safety was analyzed and qualitatively reported.

## Results

Three retrospective case series (7–9), two retrospective (10, 11), and five prospective studies (12–16) were included in our systematic review. The baseline characteristics of the patients included in the analysis are reported in Table 1. Overall, a total of 80 patients were enrolled in 10 studies: the largest study included 19 patients and the smallest included 3 patients. Most of patients were male (86%), LVEF was <35% in most patients, and 58.7% of them had ischemic cardiomyopathy. 90% of patients were treated with a radiation dose of 25 Gy using the Cyberknife system in 18.7% of patients.

**TABLE 1:** Characteristics of the studies included in the analysis.

	Robinson et al.	Cuculich et al.	Gianni et al.	Neuwirth et al.	Lloyd et al.	Carbucicchio et al.	Lee et al.	Qian et al.	Chin et al	Yugo et al.
Study design	Prospective Single-center	Case series Single-center	Prospective 2-centers	Case series Single-center	Retrospective Single-center	Prospective Single-center	Prospective 3-center	Prospective Single-center	Retrospective Single-center	Case series Single-center
No patients	19	5	5	10	10	7	7	6	8	3
Male–no (%)	17 (89.5)	4 (80)	5 (100)	9 (90)	7 (70)	7 (100)	4 (57)	6 (100)	8 (100)	2 (69)
Age	66 (49–81)	66 (60–83)	62	66 (61–78)	61 (51–78)	70 ± 7	60–70s	72 (70–73)	75 ± 7.3	72 (65–83)
Ischemic cardiomyopathy–no (%)	11 (57.9)	2 (40)	4 (80)	8 (80)	4 (40)	3 (43)	5 (71.4)	6 (100)	4 (50)	0
Non-ischemic cardiomyopathy–no (%)	8 (42.1)	3 (60)	1 (20)	2 (20)	6 (60)	4 (57)	2 (28.6)	0	4 (50)	3 (100)
LVEF (%)	25 (15–58)	23 (15–37)	34	26.5 ± 3.2	/	27 ± 11	27	20 (16–25)	21 ± 7	20–59
NYHA cl. (%)					/					
I	5.3		20			29		\		69
II	21.1		80	60		71	42.8	\		33
III	52.6	20		40			42.8	\	62.5	
IV	21.1	80					14.3	\	37.5	
Radiation type	Linac	Linac	Cyberknife	Cyberknife	Linac	Linac	Linac	Linac	Linac	Linac
Dose (Gy)	25	25	25	25	25	25	25	25	22.2 ± 3.6	25
Treatment time (min)	15.3 (5.4–32.3)	14	82 (66–92)	68 (45–80)	/	31 ± 6	38	13.8 (11–15)	18.2 ± 6	/
Mean follow up	6 months	12 months	12 ± 2 months	28 (16–54) months	5.8 (3.9–9.3) months	4 pt complete 6 months FU	6 months	7.7 (7.06–10.37) months	7.8 (4.83–9.97) months	0.5–13.5 months
VT burden reduction	94%	99.9%	No reduction	87.6%	69%	93%	85%	31%	80%	61%
Complication relater to STAR	1 pericarditis 1 heart failure (possible)	1 stroke (non-clearly related)	None	1 nausea 1 progression of mitral regurgitation	2 pneumonitis	1 nausea/vomiting 1 pulmonary fibrosis	None	1 pneumonitis 1 heart failure 1 moderate pericardial effusion	None	None

### Outcomes

Most of the studies reported a blanking period ranging between 2 and 4 months to evaluate the effectiveness of the procedure, to allow time for the formation of fibrosis. However, in a case report by Jumeau et al. (17), STAR immediately controlled an ES in a patient sedated and intubated: this suggest that radioablation may also modulate the arrhythmogenicity of the myocardial substrate with immediate mechanisms, not involving the fibrous substitution of slow conducting myocyte bundles. Most patients had fewer ventricular arrhythmias and ICD shocks after the procedure, mostly after the first month.

The duration of follow-up in these studies ranged from 6 to 54 months. The most common primary outcome was the reduction of sustained VT burden and ICD therapies (ICD shocks and ATPs). In 6 studies, there was a significant reduction (>80%) of VT burden at follow-up. In other studies reduction of VT burden at follow up ranges between 30 and 70% (9, 10, 16), or there was no reduction in one case (13).

In the first 6–12 months after treatment, almost all patients experienced recurrences of VTs or ICD shocks. Robinson et al. reported a 94% reduction in VT burden in 19 patients treated with STAR, with 89% overall survival after at 6 months (12). Cuculich et al. performed STAR on 5 patients, with a 99.9% reduction in VT burden after the first 6 months of follow-up (7).

### Adverse Effects

All the studies included reports of adverse events related to radiotherapy. Overall, two patients experienced episodes of nausea and vomiting due to the proximity of the target volume to the stomach; the symptoms were effectively managed with antiemetic therapy (13, 14). Three patients had pneumonia, and two patients developed pericarditis after radiotherapy. There was one case of progression of mitral regurgitation at the end of follow up. One patient experience stroke and two patients an episode of heart failure, but both of them were not clearly related to radiotherapy. No reductions in LVEF neither ICD malfunctions were reported. Carbucicchio et al. reported a case of paramediastinal fibrosis, which was not judged clinically relevant (14). The death of 28 (35%) patients at follow up were attributed to advanced heart failure, or non-cardiac cause rather than being directly related to radioablation treatment.

## Discussion

Stereotactic arrhythmia radioablation can be considered for patients with structural heart disease who have recurrent VT or electrical storm despite optimal antiarrhythmic drug therapy and prior CA, or in case of contraindications to CA, such as in case of aortic and mitral mechanical prosthetic valves (Table 2).

**TABLE 2:** Patients characteristic eligible to STAR and possible complications.

Patients characteristic	Complications
- Structural heart disease - Recurrent monomorphic ventricular tachycardia or electrical storm - Optimal antiarrhythmic therapy - Previous attempt of catheter ablation or contraindication	- Pneumonitis - Nausea/vomiting - Heart failure - Pericarditis/pericardial effusion - Stroke

Recurrences after CA are frequent due to the possible evolution of the substrate over time and to the three-dimensional complexity of the arrhythmia circuitry, which often extends deep inside the myocardial wall and cannot by adequately ablated with neither an endocardial nor an epicardial approach (18). In these cases, novel approaches for intramural ablation, such as needle ablation and coil embolization, can be used (19–21). In addition, bipolar ablation, in which two catheters are positioned on opposite sides of the ventricular wall has also been reported to be successful (22). However, all these techniques are currently investigational, and carry theoretical risks of serious complications. Recently, a new kind of energy called pulsed field ablation has been introduced: the ablation is non-thermally produced by creating nanoscale pores in cell membranes and has the advantage of being myocardium specific, thus minimizing collateral damage (23). Nonetheless, this energy source has not yet been tested in the ventricular myocardium in human subjects to date. It has to be mentioned that in patients who have both mechanical aortic and mitral prostheses, it is not possible to advance ablation catheters into the left ventricular endocardium with conventional routes: although there are few cases described with trans-right atrial access to the left ventricle (24), these patients are generally considered ineligible for endocardial left ventricular CA. The histological and clinical effects of ionizing radiation in tissues can appear in the weeks, months, or years after treatment (25). In the acute phase, the damage produced by oxygen free radicals causes cell necrosis with consequent tissue edema and triggering of inflammation mechanisms that eventually lead to the deposition of collagen and the formation of fibrous tissue over time. As for the treatment of cardiac arrhythmias, necrosis and consequent fibrosis may lead to the elimination of those areas of slowing of the electrical impulse propagation, which are the substrate of macroreentrant ventricular arrhythmias. Depending on the tissue involved and the replicative cellular activity, radiation energy may have different side effects (25). Clinically relevant effects of radiation on the heart involve coronary arteries, pericardium, conduction system, and valves. Radiation-induced coronary damage is characterized by sclerosis of the vascular wall, which can lead to myocardial infarction, often silent due to concomitant nerve damage. With regard to valve damage, cusps and leaflets undergo fibrotic degeneration and thickening, often associated to calcification. Pericardial damage may manifest as acute pericarditis and pericardial effusion, up to constrictive pericarditis when the resulting fibrosis impedes normal diastolic function. Although uncommon, alterations in cardiac conduction are may lead to symptomatic bradiarrhythmias and require pacemaker implantation (26). Therefore, it is clear that stereotactic radioablation carries a significant risk of clinically relevant collateral damage to the heart. The first stereotactic radioablation in humans was performed by Loo et al. for the treatment of a patient with malignant ventricular arrhythmias unresponsive to CA (27). Cuculich et al. reported the first case series comprising five patients with prior failed CA. The procedure was highly effective, with a 99.9% reduction of the burden of VT after the first 6 weeks after stereotactic radioablation (blanking period) as compared to baseline, and the risk of adverse events was low, with no complication during the index hospitalization and only one patient dying for stroke (7). Robinson et al. (12) published the largest cohort of patients treated with stereotactic arrhythmia radioablation (STAR). Nineteen patients were included in the study, two of whom with premature ventricular complexes-induced cardiomyopathy. The study showed a significant reduction in VT episodes and PVC burden, as well as a reduction of antiarrhythmic drugs (12).

Recent reviews (28, 29) showed that most of the patients treated with STAR had multiple recurrences of VT and one or more previous attempts of CA before treatment. As shown in Table 1, most of case series included patients with severe reduction of left ventricular ejection fraction and with III-IV NYHA class. Currently there is variability in patients with indication to STAR: Carbucicchio et al. in STRA-MI-VT study excluded patients in NYHA IV class, whereas Lloyd et al. in a recent case series included patients with advanced heart failure, three patients had left ventricular assist devices, and one patient had intra-aortic balloon pump support at the time of treatment (10, 14). This technique is therefore used as a last line of treatment for patients with malignant ventricular arrhythmias who do not respond to all other treatments as per current guidelines and recommendations (1, 29). Patients treated with STAR generally have advanced heart disease with a short life expectancy, in which STAR is usually considered on an individual case-by-case compassionate use basis (30, 31). Overall all studies have shown that STAR is a safe technique with low risk of serious complications in the short-to-midterm as shown in Table 2. However, it should be considered that the patients currently treated with this technique are heterogeneous due to structural heart disease and the localization of the fibrotic tissue on which the treatment is performed is variable. This could explain the variation in terms of clinical response to treatment that is observed in the various studies. Being a new and recently introduced technique, with the few data available it is not possible to correlate the variability of the clinical response to the type of structural heart disease of the patient or to the different myocardial localization of the scar. In term of safety, in the studies present in the literature there are data only in the short-to-midterm and the adverse events observed at follow up are not directly related to the treatment, considering that mainly patients with a short life expectancy being treated.

### Procedural Technical Aspect

**FIGURE 2:**
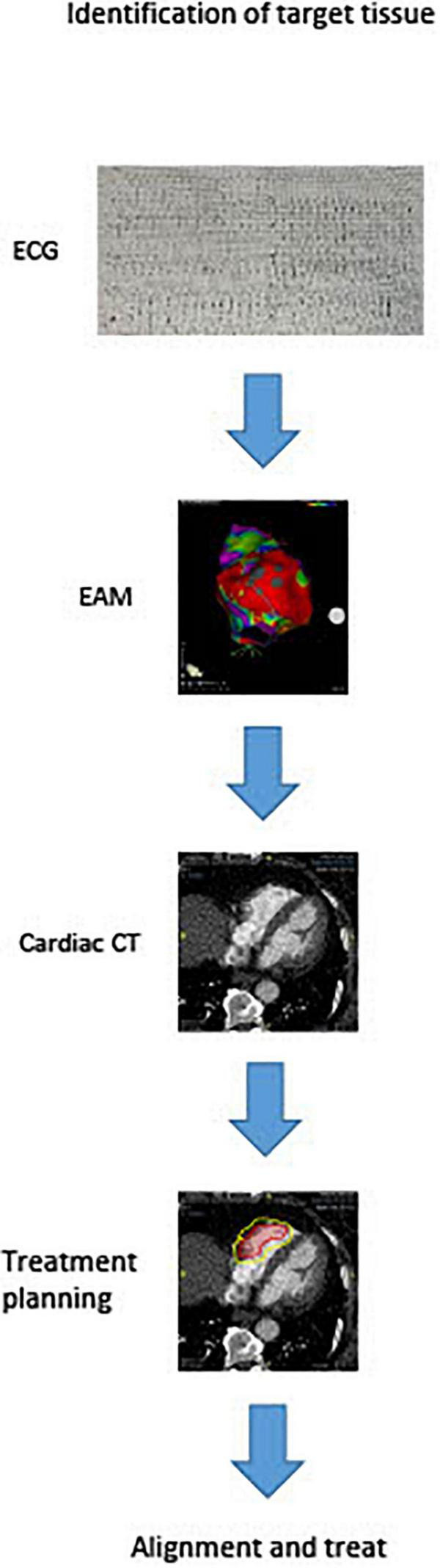
STAR technique planning.

There are currently numerous non-invasive techniques for identifying scar areas from which ventricular tachycardias are arising. These include cardiac MRI and CT scan. Recently, Soto-Iglesias et al. (32) established the feasibility of VT CA guided by cardiac magnetic resonance (CMR), using pixel signal intensity (PSI) maps derived from late gadolinium enhancement (LGE) CMR sequences. This approach enables a totally non-invasive identification of the target area to be treated, and may soon enter the clinical arena for STAR planning. Actually studies report that the arrhythmogenic target for the ablation can be identified by substrate map using electroanatomic mapping to create a three-dimensional map in order to locate scar tissue and the target of the radioablation. After then the anatomical target for radioablation is defined with cardiac computed tomography (CT) as shown in Figure 2. Treatment radiation planning is then created upon this technical information. This anatomical portion of the heart is will be used to center the radiation dose just before treatment. Cooperation between different professionals for the treatment of VTs refractory to drug therapy and catheter ablation is key for STAR. Clinical indication is decided primarily by the cardiac electrophysiologist who identifies patients with structural cadiomiopathy and ventricular arrhythmias that could be eligible for treatment and performs an electroanatomical mapping. The radiologist with experience in cardiac imaging and the radiotherapist will subsequently evaluate the technical possibility of the STAR and its planning. Almost all studies used a single dose of 25 Gy for STAR and treatment time was variable, ranging from few minutes to thousand minutes. This range of treatment duration varies from different types of groups (8, 13, 14, 33). However, this dose was in some cases reduced, when the target volume was localized in the diaphragmatic face of the heart. In these cases, in which the region of interest was located in the inferior wall of the left ventricle, multiple doses with deep inspiration-breath hold to reduce radiation dose to the stomach can be effective (34).

## Conclusion

Actually, there are few studies in literature, which included small patient populations. The treatment involves various professional figures (cardiologists, electrophysiologists, radiologists, and radiotherapists), and is not yet standardized, varying with the experience of the center. STAR currently appears to be effective in reducing the burden of ventricular arrhythmias and ICD treatments, with no serious adverse effects in the short-to-midterm directly related to STAR. However, more studies involving a larger sample population are necessary to improve the efficacy and safety of treatment. Longer follow up are also needed to assess the safety of the treatment.

## Data Availability Statement

The original contributions presented in this study are included in the article/supplementary material, further inquiries can be directed to the corresponding author.

## Author Contributions

GV, PC, and MC: conceptualization and resources. LCi, QP, YV, LCa, AG, AD, and MC: writing—review and editing. MC and AD: supervision and project administration. All authors contributed to writing—original draft preparation and read and agreed to the published version of the manuscript.

## Conflict of Interest

The authors declare that the research was conducted in the absence of any commercial or financial relationships that could be construed as a potential conflict of interest.

## Publisher’s Note

All claims expressed in this article are solely those of the authors and do not necessarily represent those of their affiliated organizations, or those of the publisher, the editors and the reviewers. Any product that may be evaluated in this article, or claim that may be made by its manufacturer, is not guaranteed or endorsed by the publisher.
